# C- and N-truncated antimicrobial peptides from LFampin 265 – 284: Biophysical versus microbiology results

**DOI:** 10.4103/0975-7406.76467

**Published:** 2011

**Authors:** Regina Adão, Kamran Nazmi, Jan G.M. Bolscher, Margarida Bastos

**Affiliations:** Department of Chemistry and Biochemistry, Centro de Investigação em Química-CIQ(UP), University of Porto, Portugal; 1 Department of Oral Biochemistry, Academic Center for Dentistry Amsterdam (ACTA), Amsterdam, The Netherlands

**Keywords:** Activity assays, antimicrobial peptides, CD, DSC, lactoferrin derived peptides, peptide / membrane interaction

## Abstract

Lactoferrin is a glycoprotein with two globular lobes, each having two domains. Since the discovery of its antimicrobial properties, efforts have been made to find peptides derived from this protein showing antimicrobial properties. Most peptides initially studied were derived from Lactoferricin B, obtained from the protein by digestion with pepsin. More recently, a new family of antimicrobial peptides (AMPs) derived from Lactoferrin was discovered by Bolcher *et al*, and named Lactoferrampin (LFampin). The original sequence of LFampin contained residues 268 – 284 from the N1 domain of Lactoferrin. From this peptide, the Bolscher’s group synthesized a collection of peptides obtained by extension and / or truncation at the C or N-terminal sides, in order to unravel the main structural features responsible for antimicrobial action. Here, we present results for three of these peptides, namely LFampin 265 – 284, LFampin 265 — 280, and LFampin 270 – 284. The peptides were tested against bacteria (*E. coli* and *S. sanguinis*), fungi (*C. albicans*), and model membranes of 1,2-dimyristoyl-*sn*-glycero-3-phosphocholine (DMPC), 1,2-dimyristoyl-sn-glycero-3-[phospho-rac-(1-glycerol)] (DMPG), and their mixtures at a ratio of 3 : 1 (DMPC : DMPG (3 : 1)). The ability to adopt a helical conformation was followed by a circular dichroism (CD), and the perturbation of the *gel* to the *liquid-crystalline* phase transition of the membrane was characterized by differential scanning calorimetry (DSC). Distinct behavior was observed in the three peptides, both from the microbiology and model membrane studies, with the biophysical results showing excellent correlation with the microbiology activity studies. LFampin 265 – 284 was the most active peptide toward the tested microorganisms, and in the biophysical studies it showed the highest ability to form an α-helix and the strongest interaction with model membranes, followed by LFampin 265 – 280. LFampin 270 – 284 was inactive, showing marginal secondary structure and no interaction with the pathogen model membranes.

In recent years, a large number of studies have been directed to the evaluation of the action of antimicrobial peptide (AMP) on target pathogens, due to the huge increase in pathogenic microorganisms multiresistant to conventional antibiotics.[1–6] In this scenario, substantial efforts are being devoted to the discovery of new antibiotic resources and strategies, and antimicrobial peptides (AMPs) are receiving considerable attention as a new paradigm in antibiotic therapy. There is a general agreement that most peptides have the bacterial membrane as the main target of their antimicrobial action, which may proceed through different mechanisms, such as formation of stable pores (either barrel-stave or toroidal pore’s type), membrane thinning (molecular electroporation or sinking rafts models) or micellization of the membrane (in a detergent-like action (carpet model).[7–13] Although most AMPs have a wide range of activity, subtle differences are found with regard to different spectra of activity, which must derive from a combination of factors such as size, amino acid sequence, charge, secondary structure upon membrane interaction, hydrophobicity, hydrophobic moment, and amphipathicity. These parameters are interdependent and a change in one of them can alter the structure-activity relationship, thereby, influencing the ability of the peptide to interact with the pathogen.

Bovine Lactoferrin is a glycoprotein, composed of a polypeptide chain containing 703 amino acids folded into two globular lobes, called the C– (carboxy) and N– (amino) terminal regions, connected with an α-helix. Each lobe consists of two domains: C1 and C2, and N1 and N2. Most of the antimicrobial activity is attributed to the N1 domain, and the first group of peptides studied derived from this domain of Lactoferrin, comprising of amino acids 17 – 41, and designated as Lactoferricin B (LFcin B).[14–18] Of late, another amino acid sequence, also located in the N1 domain was identified and studied in the Bolscher’s group,[19,20] by searching for key determinants for antimicrobial activity, such as, the presence of stretches with alternating positively charged and uncharged residues with the potential to form a positively charged amphipathic α-helix.[19] The initial sequence contained amino acids 268–284, and thereafter, several peptides were synthesized, and obtained by truncation / extension of the original sequence.[21]

Among these, we chose three peptides for the present study, namely LFampin 265 – 284, LFampin 265 – 280, and LFampin 270 – 284. Taking LFampin 265 – 284 as the lead peptide, the two others were obtained by truncation on the C side (LFampin 265 – 280) and on the N side (LFampin 270 – 284), as these two shortenings mainly affected two different parameters — cutting on the C side reduced the charge, and on the N side the tendency to adopt a helical structure, as shown by van der Kraan *et al*,[21] In this manner, we attempted to discriminate the properties that were more important for antimicrobial potency and how the differences were reflected in the peptide’s interaction with the membrane. Some properties of these peptides are summarized in Table 1.

**Table 1:** Properties of synthetic LFampin peptides

Peptide	Sequence	#AA*	M_r_**	Charge^a^	*<μ>*_b_^b^	*<H>*_c_^c^
LFampin 265 – 284	DLIWKLLSKAQEKFGKNKSR	20	2389	4 +	0.30	- 0.337
LFampin 265 – 280	DLIWKLLSKAQEKFGK	16	1904	2 +	0.37	- 0.186
LFampin 270 – 284	LLSKAQEKFGKNKSR	15	1733	4 +	0.28	- 0.437

* #AA= number of amino acids in the amino acids sequence.

** M_r_ = molar mass (kDa).

a Net positive charge at neutral pH

b *<μ>;* mean hydrophobic moment in a α-helical conformation.[41].

c <*H*>; mean hydrophobicity[49]

## Materials and Methods

### Microorganisms and culture conditions

Two strains — *Streptococcus sanguinis* SK4 and *Escherichia coli* K12 — were cultured aerobically at 37°C in brain heart infusion (BHI) medium from Difco (Becton Dickinson Microbiology). Yeast *Candida albicans* 315 was cultured aerobically at 30°C in Sabouraud dextrose broth source. The microorganisms were cultured overnight and subcultured for two-to-three hours to yield a mid-logarithmic growth culture at the time of harvesting.

### Synthesis and purification of peptides

Bovine lactoferrin peptides [Table 1] from the LFampin domain were synthesized with a Milli-Gen 9050 peptide synthesizer (MilliGen/Biosearch, Bedford, MA) according to the manufacturer’s procedures. Peptides were purified to a purity of at least 95% with semi-preparative RP-HPLC (Jasco, Tokyo, Japan) on a Vydac C18-column (218MS510, Vydac, Hesperia, CA). The authenticity of the peptides was confirmed by ion trap mass spectrometry with an LCQ Deca XP (Thermo Finigan, San Jose, CA) as described previously.[21]

### Antimicrobial activity

Bactericidal and candidacidal activity of the peptides was determined by peptide-mediated membrane permeabilization, monitored by the fluorescence enhancement of propidium iodide (PI, Invitrogen, Breda, The Netherlands) in dead cells, as described previously.[22] Briefly, a mid-log phase culture of bacterial suspensions (approximately 2.5×10^8^ CFU/mL) or C. *albicans* suspension (approximately 1.5×10^7^ CFU/mL) in 96-well U-bottom low affinity plates (Greiner Bio One) were supplemented with PI (final concentration 6 mM) and incubated with equal volumes of peptide solutions at final concentrations of 0.2-50 mM, at 37°C. Fluorescence was monitored at λ_exc_ 544 nm and λ_em_ 620 nm using a fluorescence reader (Fluostar Galaxy, BMG Labtechnologies, Offenburg, Germany) with five minute time intervals till 15 minutes followed by 15 minute intervals till one hour. LC_50_ values (mM) were the concentrations of the peptides resulting in 50% killing. All experiments were repeated at least twice in duplicate.

### Preparation of liposomes

Appropriate amounts of 1,2-dimyristoyl-sn-glycero-3-phosphocholine (DMPC), 1,2-dimyristoyl-*sn*-glycero-3-[phospho-*rac*-(1-glycerol)] (DMPG) (Avanti Polar Lipids, Alabama, USA), and a DMPC:DMPG mixture at a molar ratio of 3 : 1 were dissolved in chloroform / methanol (3 : 1 v/v). The solution was dried under a slow nitrogen flow and the resulting lipid films were kept under vacuum for three hours to remove all traces of organic solvents. The lipid film was hydrated with 10 mM HEPES buffer (pH 7.4) containing 100 mM NaCl, at 10°C above the temperature of the *gel*, to *liquid crystalline* phase transition (T_m_). The resulting multilamellar vesicles (MLVs) were frozen in liquid nitrogen and thawed in a water bath at approximately 10°C above T_m_ (five cycles). Large unilamellar vesicles (LUVs) were obtained from the MLVs by extrusion in a 10 mL stainless steel extruder (Lipex Biomembranes Inc., Vancouver, Canada) and thermostated at 10°C above T_m_ . The samples were passed several times through polycarbonate filters (Whatman, Nucleopore, NJ, USA) of decreasing pore size (600, 200, and 100 nm; 5, 5, and 10 times, respectively), under inert (N_2_) atmosphere. The phospholipid concentration was determined for each preparation by the phosphomolybdate method.[23]

#### Circular dichroism

Circular Dichroism (CD) experiments were carried out in a Jasco 720 spectropolarimeter (Japan Spectroscopy Co., Tokyo) equipped with a rectangular cell, path length of 1 mm.[24] Scans were performed between 175 – 250 nm, bandwidth 1.0 nm, and resolution of 100 mdeg. Measurements using pure buffer (2 mM HEPES, 100 mM NaCl, pH 7.4) were performed throughout, to test instrument reproducibility. Spectra of pure liposome preparations in the same solvent media at different concentrations were used in a blank experiment to be subtracted from the liposome / peptide spectra. The peptide concentration in buffer and in liposome / peptide mixtures was 36 *μ*M. Liposome concentrations were: 6000 *μ*M for DMPC (with 0.6% peptide), 1200 *μ*M for DMPG (with 3% peptide), and 3000 *μ*M DMPC : DMPG (3:1) (with 0.6% peptide), where peptide percentages were in mol / mol. The peptide / lipid ratio shown corresponded to the ones for which the best spectra definition was obtained (1:167 for the DMPC and for DMPC : DMPG; and 1:36 for DMPG). The desired amounts of peptide and liposome were mixed immediately prior to each measurement and incubated at 35°C for 30 minutes before the measurements, and performed at the same temperature. Each spectrum was always the average of nine accumulations. After blank correction, the observed ellipticity was converted to a mean residue molar ellipticity (θ) (deg^.^cm^2.^dmol^−1^), based on the total amount of peptide present in the mixture.

### Differential scanning calorimetry

Differential scanning calorimetry (DSC) was performed in a Micro-DSCIII microcalorimeter (SETARAM, Caluire, France) essentially as described previously.[24] Two successive up and down scans were performed for each sample, the up-scan at a scanning rate of 0.5°C/minute and the down-scan at 3°C/minute, over the temperature range 10 – 35 °C. The sample mixtures were prepared immediately before the DSC run, by adding the desired amount of peptide (LFampin 265 – 284, LFampin 265 – 280 or LFampin 270 – 284) stock solution to the LUVs suspension of DMPC, DMPG or DMPC : DMPG (3 : 1). Samples with 0.5, 0.75, 1.0, 2.0, and 3.0% (mol/mol) were used. All procedures regarding sample preparation and handling (lag time at low temperature, time between mixtures, and start of the experiment) were kept constant in all experiments, to ensure that all samples had the same thermal history. The instrument was electrically calibrated for temperature and the scan rate with the SETARAM Calibration Unit.[25] The Micro-DSCIII software was used for baseline subtraction (run with buffer solution on both cells (sample and reference)). The transition temperature Tm and the transition enthalpy change (∆_trans_*H*) were calculated by integration of the heat capacity versus temperature curve (Cp *versus* Temperature). A linear baseline was used to calculate the integral areas under the curves.[24,26,27]

## Results and Discussion

### Bactericidal and candidacidal activity of LFampin peptides

The lead antimicrobial peptide LFampin 265 – 284 comprises of a highly cationic C-terminal part and an α-helix facilitating N-terminal part.[21] To analyze the impact of either part on the antimicrobial activity of the bovine lactoferrin antimicrobial peptide LFampin 265 – 284, its behavior was compared with two peptides truncated at either the N- or C-terminus of the LFampin 270 – 284 and LFampin 265 – 280, respectively. For representative target microorganisms, we used Gram-negative *Escherichia coli*, a rather harmless indigene of the lower intestine (although some strains could cause serious food poisoning in humans),[28] the Gram-positive *Streptococcus sanguinis*, which was a normal inhabitant of the healthy human mouth (although if it gained entrance into the bloodstream it was the most common cause of subacute bacterial endocarditis),[29] and the yeast *Candida albicans* which was an opportunistic pathogen and the causal agent of oral and genital infections in immunocompromised persons e.g., in AIDS, cancer chemotherapy, and transplantation patients.[30]

**Figure 1:**
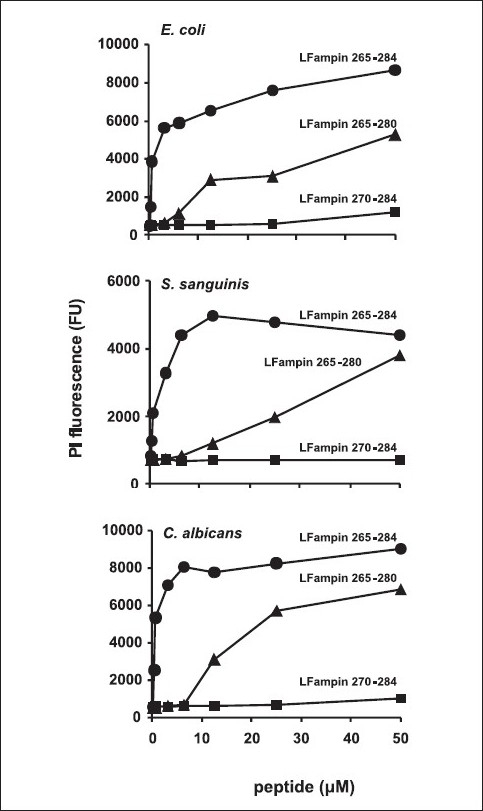
Antimicrobial activity of LFampin peptides. Microorganisms were incubated with two-fold serially diluted peptides. Graphs represent fluorescence uptake after 1h of incubation

When compared with the leading and most active peptide LFampin 265 - 284, although LFampin 270 – 284 was found to be completely inactive, LFampin 265 – 280 retained some activity toward the tested microorganisms. The levels of antimicrobial activities found with 50 *μ*M of LFampin 265 – 280 were reached with less than 3 or 6 *μ*M LFampin against *C. albicans, E. coli*, and *S. sanguinis*, respectively [Figure 1]. Similar graphs were already found within 10 minutes of incubation and remained unchanged thereafter (not shown).

Secondary structure of LFampin 265 – 284, LFampin 265 – 280, and LFampin 270 – 284 peptides in buffer solution and in the presence of membranes, as studied by CD

**Figure 2:**
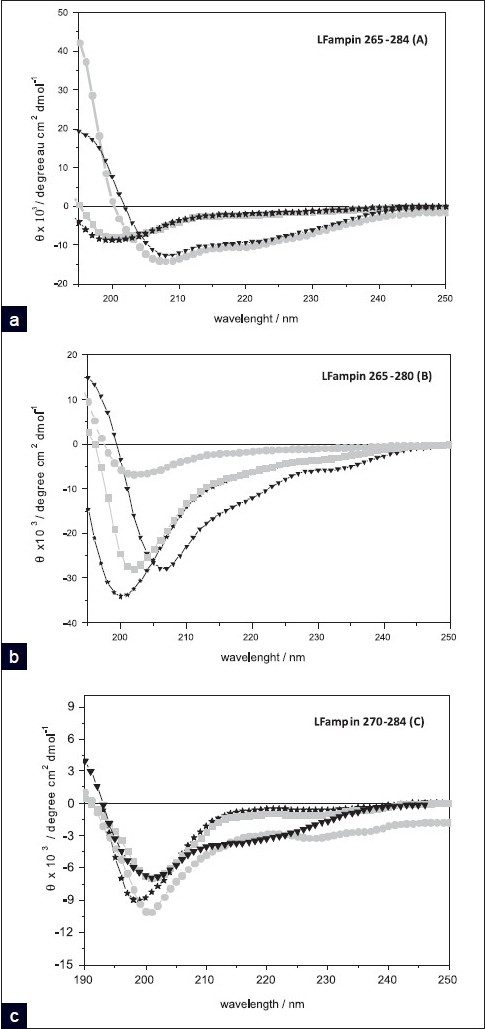
CD spectra of LFampin 265-284 (a), LFampin 265-280 (b) and LFampin 270–284 (c) Buffer (36 μM peptide) (black stars); 6000 μM DPMC and 0.6% peptide (light gray squares); 1200 μM DMPG and 3% peptide (black triangles); in 3000 μM DMPC:DMPG (3:1) and 0.6% peptide (light gray circles)

Indication of secondary structures of the three peptides was obtained by CD spectra, Figures 2a–c. It can be seen that in Hepes buffer (2 mM and 100 mM NaCl) all peptides show a minimum, around 200 nm, characteristic of a random structure.

When in the presence of membranes, a different secondary structure is acquired, depending on the peptide and the nature of the model membrane. The CD spectrum of the lead peptide LFampin 265 – 284, in the presence of DMPC, overlaps the one observed in the buffer, indicating no significant change of structure in the presence of liposomes for all concentrations tested (1200 – 6000 mM, results showed only to 6000 mM). The other two peptides, LFampin 265 – 280 and LFampin 270 – 284, show a slight shift from 200 nm to the right, indicating that a small change of structure could be present, but without formation of the a well-defined different secondary structure. It must be noted that the peptides LFampin 265 – 280 and LFampin 270 – 284 in buffer do not present a CD spectra compatible with a pure random structure (as LFampin 265 – 284 does), indicating that a mixture of structures is present. Therefore, the small shift cannot be over-interpreted, and thus there are no significant changes of secondary structure for any of the three peptides in the presence of DMPC membranes.

In the presence of liposomes of DMPC : DMPG (3 : 1) (here used as model for pathogens) LFampin 265 – 284 forms an α-helix structure, reflected in the two minima at wavelengths near 208 and 222 nm. LFampin 270 – 284 presents a spectra close to the one obtained in the buffer, whereas, for LFampin 265 – 280 some indication of an α-helix is apparent, although to quite a small extent (note that the obtained signal is always a weighted mixture of all the structures present) [Figure 2].

In order to evaluate the importance of charge effects and to compare them with the results we previously obtained for LFampin 265 – 284 in pure DMPG membranes,[22] we did also study the truncated versions in the presence of this model membrane system. We could see [Figure 2] that LFampin 265 – 284 also formed an α-helix in this membrane system [Figure 2a], perfectly super imposable with the spectra in DMPC : DMPG (3:1) discussed earlier, whereas, LFampin 265–280 clearly showed the presence of some α-helix structure (albeit to a smaller extent, for the same P:L ratio), and finally LFampin 270 – 284 showed a shift in minima, but no clear α-helix structure. The fact that a helix was found for LFampin 265 – 280 when the model membrane system was totally formed by the negatively charged DMPG, showed the importance of the membrane charge combined with the charge and amphipathic character of the peptide, as the presence of DMPC in the mixed membranes caused a charge distribution on the surface of the liposome, and thus a larger amount of peptide was necessary to induce the same amount of secondary structure for this peptide.

In order to quantify the amount of each structure present, we calculated the percentage of the α-helix, β sheet, and randomized structures for each peptide in the presence of the three membrane systems. The percentages of each structure were calculated by fitting a linear weighted sum of structures, as proposed by Chen,[31] to the mean molar ellipticity per amino acid residue, using the Solver facility in Excel (Microsoft^TM^). The reference values for the average ellipticity of each structure were those provided by Greenfield and Fastman[32] based on synthetic polypeptides. The Excel sheet for this calculation was developed by us based on the references and values referred to, as most available software for these calculations was developed for proteins, and we found that this approach provided much better estimates. The obtained values can be seen in Table 2. The results show, as expected from the spectra, that all peptides are still predominantly random in the presence of DMPC membranes, although LFampin 270 – 284 presents a significant percentage of β sheet structure.

**Table 2:** Contribution (in %) of each secondary structure, α-helix, β-sheet, and random structure, to the total CD signal, calculated for each peptide by fitting procedures as described in the text

Peptides	DMPC			DMPG			DMPC:DMPG (3:1)		
	α	β	random	α	β	random	α	β	random
LFampin 265 – 284	25	20	55	50	10	40	50	25	25
LFampin 265 – 280	32	4	64	55	0	45	40	0	60
LFampin 270 – 284	5	43	52	19	22	59	9	37	54

* The estimated uncertainties from the fittings in the reported values are ± 2

LFampin 265 – 284 and LFampin 265 – 280 show the highest percentage of α-helix (50 and 55%, respectively) in the presence of DMPG liposomes and that percentage is the same for LFampin 265 – 284, in the presence of DMPC : DMPG (3 : 1), whereas for LFampin 265 – 280 it is lower. This finding emphasizes the importance of using mixed membranes to simulate pathogen membranes (rather than purely negative membranes), as its use led us to differentiate between these two peptides’ effects. LFampin 270 – 284, on the other hand, still presents a higher percentage of random structure in all membrane systems studied.

**Figure 3:**
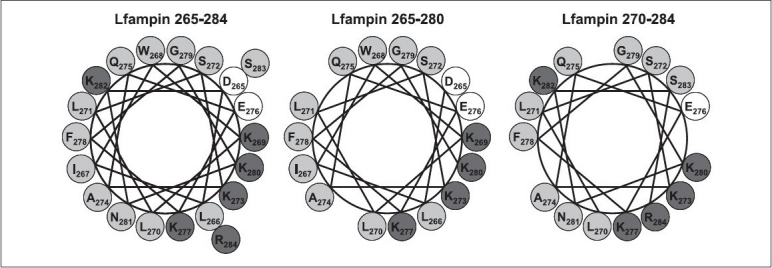
Helical wheel presentation of LFampin peptides, showings their amphipatic character. Neutral amino acids: light grey circles; positively charged amino acids: dark grey and negatively charged amino acids: white

Indeed the peptide to lipid ratio also affects the formation of a secondary structure, as the structure formation is dependent on partitioning, and the amount of peptide in the membrane changes with the P : L ratio. The spectra presented were obtained for the most suitable P : L ratio, to produce a good signal in the CD spectra. The observed differences for the three peptides reflect their change in structure, and their strength relates to the degree of partition. The removal of the amino acid lysine (K) and arginine (R) (positively charged) and asparagine (N) and serine (S) (both polar) of the C-terminus side (from LFampin 265 – 284 to LFampin 265 – 280), decreased the peptide charge from + 4 to + 2, as well as its polarity, thus affecting the tendency to form an α-helix [Figure 3]. The absence of amino acids N, K, S, and R increased the hydrophobic moment (0.30 for LFampin 265 – 284 and 0.37 for LFampin 265 – 280) and increased the peptide hydrophobicity (- 0.337 for the LFampin 265 – 284 and - 0.186 for the LFampin 265 – 280) [Table 1]. These values suggest a hydrophobic / hydrophilic balance more suitable for a good interaction between DMPG liposomes and the LFampin 265 – 284 peptide. Moreover, the truncation of the amino acids sequence on the N-terminal side (from LFampin265 – 284 to LFampin 270 – 284) [Figure 3], with the removal of the amino acids aspartic acid (D) (residue 265, charged negatively), leucine (L) (residue 266, nonpolar), isoleucine (I) (residue 267, nonpolar), tryptophan (W) (268 residue, polar), and lysine (K) (269 residue, charged positively) did not change the charge of the peptide (+ 4), but decreased the hydrophobic moment (from 0.30 to 0.28 for LFampin 265 – 284 and LFampin 270 – 284, respectively) and decreased the hydrophobicity (from - 0.337 to - 0.437 for LFampin 265 – 284 and LFampin 270 – 284, respectively), which was reflected in an almost nonexistent tendency in LFampin 270 – 284 to form the α-helix and partition into the membranes. Furthermore, the lack of tryptophan in LFampin 270 – 284 was also important in the reduction of the partition, as this amino acid was often considered to have an important role in ‘anchoring’ the peptide in the membrane.[13,33]

The CD results for the studied peptides are consistent with the literature results. NMR studies using negatively charged SDS micelles (sodium dodecyl sulfate) and zwitterionic DPC (dodecylphosphocholine) liposomes with the LFampin 268 – 284 peptide (the original sequence of LFampin), showed that the peptide formed an α-helix involving only residues 1 – 11 and the final six C-terminal residues remained relatively unstructured.[34] We observed a well-defined α-helix for the LFampin 265 – 284 in DMPG and DMPC : DMPG liposomes, confirming the influence of the amino acids aspartic acid (D), leucine (L), and isoleucine (I) in the formation of the secondary structure.

The secondary structures of LFampin 265 – 284, LFampin 265–280, and LFampin 270–284 were determined in the presence of trifluoroethanol / water (TFE / water) by van der Kraan *et al*,[21] In this study the authors found that the three peptides were able to form an α-helix, but to a different extent: LFampin 265 – 284 showed the highest tendency to form an α-helix, followed by LFampin 265 – 280, and finally LFampin 270 – 284. The residual tendency of LFampin 270 – 284 to form an α-helix in helix-inducing solvents was not apparent in the presence of membranes (at the P:L ratios used), and this reinforced the need for these studies to be performed with membranes, when a biological correlation was aimed at. Moreover, studies with mimetic membrane allowed the differentiation of the secondary structures formed and their dependence on the composition of the membrane, which was a fundamental aspect in the possible biological implications.

### Interaction of the peptides with liposomes as studied by differential scanning calorimetry

The thermodynamic characterization of peptide / liposome interactions by DSC is based on the changes in the thermal profile and in the thermodynamic parameters characterizing the thermally induced transitions in liposome systems (T_m_ and ∆_trans_*H*), due to the presence of the peptides.

**Figure 4:**
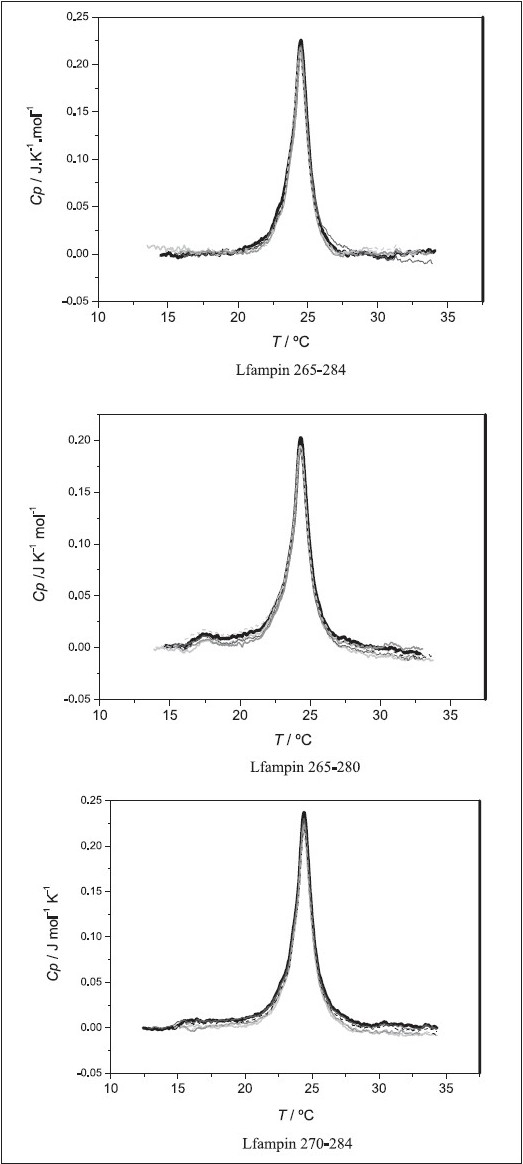
DSC curves for DMPC liposomes and the studied peptides at different peptide molar ratios. Black: pure DMPC; light gray: 0.5 mol% peptide; dark gray: 0.75 mol% peptide; gray: 1.0 mol% peptide; dash black: 2.0 mol% peptide; dash light gray: 3.0 mol% peptide. Lipid concentration was in all cases 3 mM

The DSC curves of pure DMPC liposomes (LUVs) and peptide / lipid mixtures at different P : L ratios are shown in Figure 4. The derived phase transition temperature values T_m_ and change in transition enthalpy ∆_trans_*H* are reported in Table 3. In all cases, we see that the peptide affects neither the thermal profile nor the derived thermodynamic parameters for the gel to liquid-crystalline transition of DMPC liposomes [Figure 4]. We can thus conclude that for the studied P : L ratios, none of the peptides partition to the zwitterionic membranes. The pre-transition is observed when MLVs are used (temperatures between 16 and 17.5°C)[35,36] are usually not so clear with LUVs, as it appears superimposed with the main transtition.[37] Its change is not discriminatory, as it disappears in the presence of most added drugs,[35,38–41] hence, we have not deconvoluted the two transitions and will not address it here.

**Table 3:** Transition temperature) Tm and transition enthalpy change ∆trans*H* values for the three peptides in the three model membrane systems studies, DMPC, DMPG, and DMPC : DMPG (3 : 1) liposomes. The results are presented as a function of peptide % (or P : L ratio)

*T_m_ (°C) / ∆_trans_*H* (kJ mol^-1^)*										
% Peptide	(P:L)	DMPC			DMPG			DMPC:DMPG (3:1)		
		265-284	265-280	270-284	265-284	265-280	270-284	265-284	265-280	270-284
0	0	24.5/19	24.3/20	24.4/21	23.4/23	23.9/23	23.3/24	25.0/21	24.9/22	24.8/22
0	0	24.5/19	24.3/20	24.4/21	23.4/23	23.9/23	23.3/24	25.0/21	24.9/22	24.8/22
0.50	1:196	24.5/21	24.3/21	24.4/20	23.1/25	23.4/21	23.2/24	24.9/21	24.8/20	24.9/22
0.75	1:129	24.5/21	24.3/19	24.4/20	23.0/21	23.3/20	23.1/24	24.9/19	24.8/19	24.8/21
1.0	1:96	24.5/19	24.3/19	24.4/20	23.0/20	22.9/17	22.9/24	24.8/19	24.9/21	24.8/21
2.0	1:46	24.5/18	24.3/20	24.4/19	21.0/17	25.4/20	22.4/22	24.4/20	24.8/22	24.8/21
3.0	1:29	24.4/18	24.3/20	24.4/19	-	25.5/17	21.9/22	23.9/18	24.6/22	24.8/20

Estimated uncertainties: within sample is ± 0.1°C for T_m_ and ± 0.5 kJ mol^-1^ for ∆_trans_*H*; and between samples is ± 0.3°C for T_m_ and ± 3 kJ mol^-1^ for ∆trans*H*

**Figure 5:**
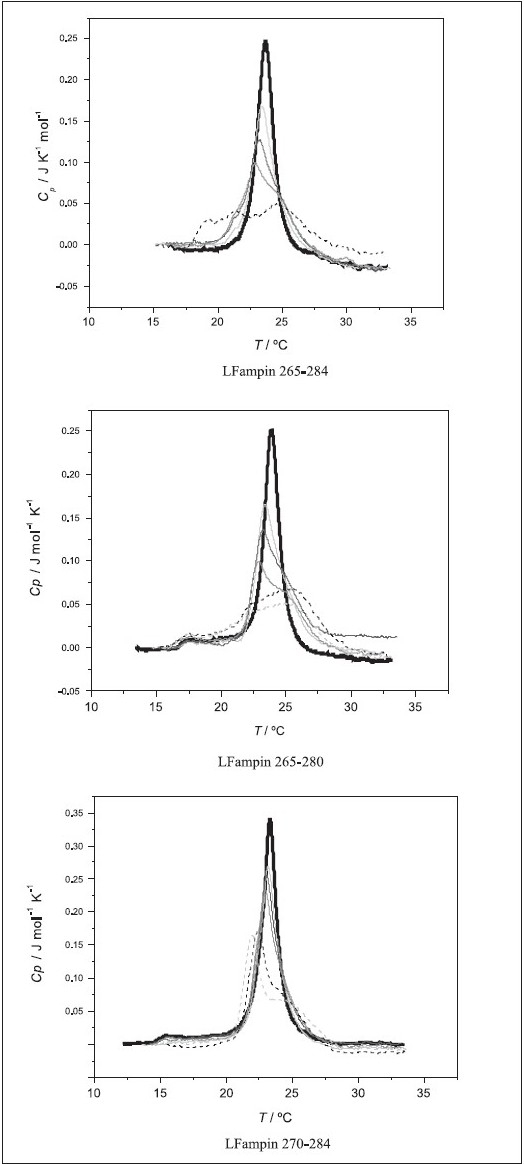
DSC curves for DMPG liposomes and the studied peptides at different peptide molar ratios. Black: pure DMPG; light gray: 0.5 mol% peptide; dark gray: 0.75 mol% peptide; gray: 1.0 mol% peptide; dash black: 2.0 mol% peptide; dash light gray: 3.0 mol% peptide. Lipid concentration was in all cases 3 mM

The DSC curves obtained in the presence of DMPG liposomes at different P : L ratios are shown in the Figure 5 and the respective thermodynamic parameters are reported in Table 3. In this case the peptides alter both the profiles, the T_m_ and the ∆_trans_*H*(at the highest P : L ratios). Also here, the largest changes are observed for LFampin 265 – 284[22] and LFampin 265 – 280, and the smallest for LFampin 270 – 284. At the highest percentage of peptide, 2 mol% (P : L = 1 : 46) and 3 mol% (P : L = 1:29), the ∆_trans_*H* decreased for the first two peptides, whereas, the parameters were the same (within uncertainty limits) for the later one. Furthermore, a broadening of the curves (decrease in cooperativity) and the appearance of a shoulder at higher temperatures was also observed in all the cases. Finally, it should be noted that for LFampin 265–284 and LFampin 265–280 the transition was totally distorted at the highest ratios, whereas, LFampin 270–284 still presented a transition curve, again showing that this peptide had the weakest interaction.

The shoulder at higher temperatures can be interpreted as reflecting the electrostatic attraction between the peptide and the lipid head, leading to a stabilization of the gel phase. Precipitation / aggregation was observed for the three peptides at the highest P : L ratios (2 and 3%).

**Figure 6:**
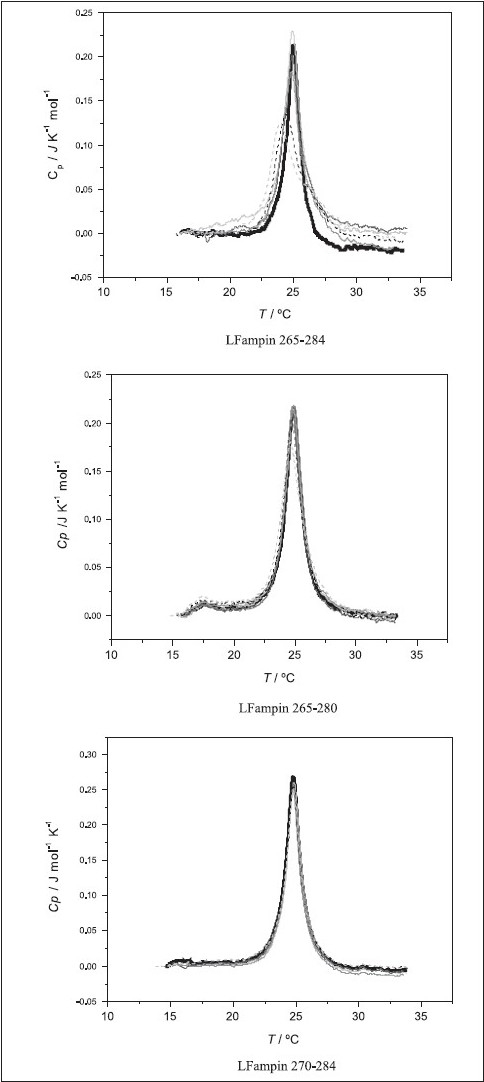
DSC curves for DMPC:DMPG (3:1) liposomes and the studied peptides at different peptide molar ratios. Black: pure DMPC:DMPG; light gray: with 0.5 mol% peptide; dark gray: with 0.75 mol% peptide; gray: with 1.0 mol% peptide; dash black: with 2.0 mol% peptide; dash light gray: with 3.0 mol% peptide. Lipid concentration was in all cases 3 mM

Finally the results for DMPC : DMPG (3 : 1) can be seen in Figure 6. For the studied P : L ratios, neither LFampin 265 – 280 nor LFampin 270 – 284 show significant changes in the DSC profile or parameters. The thermotropic behavior shown with LFampin 265 – 284 is intermediate between the one observed for the zwitterionic DMPC and the anionic DMPG liposomes, and both Tm and ∆_trans_*H* decrease gradually as the P : L ratio increases Table 3. At the highest P : L ratio a shoulder at higher temperatures is apparent, which is a strong indication of lipid segregation within the membrane, due to the preferential interaction of the peptide with the negatively charged lipid DMPG. Prenner *et al*,[42] also observed a more strong interaction with anionic DMPG than zwitterionic DMPC or DMPE phospholipid bilayers, with the cationic peptide gramicidin S (GS), due to electrostatic effects. At higher peptide concentrations, gramicidin S (GS) reduced the temperature, enthalpy, and cooperativity of the main phase transition in the DMPG bilayers. Furthermore, a decrease in cooperativity in the phase transition is observed when DMPG liposomes are involved, further substantiating a better interaction and higher disturbance of membrane patches, richer in DMPG when the mixed DMPC / DMPG system is used. The decrease in the transition enthalpy at higher P : L ratios is compatible with the partial insertion of the peptide in the lipid bilayer, which causes a change in the packaging of carbon chains, by disruption of the van der Waals inter- and intrα-molecular interactions.[24]

The analysis of the thermotropic profile of liposomes of different compositions in the presence of peptides, allows us to conclude that the interaction between antimicrobial peptides and lipid bilayers involves factors related to the characteristics of the peptide and also to the lipid composition of the membrane. The DSC data reflect the low affinity of these peptides to DMPC liposomes, and this is confirmed by our CD results where the peptides remain unstructured in the presence of this membrane system. Finally, the results obtained by DSC confirm that the presence of anionic lipid boosts the action of the peptide by the initial electrostatic interactions, facilitating peptide insertion and membrane destabilization.

The characteristics of the peptides responsible for the distinct behavior include: the peptide charge, the tendency to form α-helix in the presence of the membrane, and the amphipathicity of the helix formed. The three peptides are capable of electrostatic interactions with the negatively charged membranes because all have a positive charge. LFampin 265 – 284 and LFampin 270 – 284 have the same nominal charge (+4) and LFampin 265 – 280 has the lowest (+2), but nevertheless it disturbs DMPG to a much larger extent than LFampin 270 – 284. This confirms that the charge alone is not the key factor in differentiating the effect of peptides on membranes and our results show that the secondary structure formed in their presence is a more important factor for a larger partition and consequently a more effective interaction. CD results [Figure 2] show that the LFampin 265 – 284 forms the highest percentage of α-helix, followed by LFampin265 – 280. In Figure 3 it can be seen that LFampin 265 – 284 has a helical structure, with the positive charges more clustered on one side of the helix, thus forming a more amphipathic α-helix, more suited to interact with the polar heads of the lipid bilayer. Thus, although both effects (charge and secondary structure) are important, the results indicate that the absolute charge is less significant in an effective interaction with the membranes, than the ability to form a well-defined secondary structure.

The interaction of the original sequence of the LFampin peptide (residues 268 – 284) with the multilamellar liposomes (MLVs) of DPPC and DPPG was studied by DSC, by Vogel and colleagues.[34] The results of this study showed that the peptide did not affect the thermotropic profile of zwitterionic liposomes (DPPC) across the range of the studied P : L ratios. In anionic liposomes (DPPG) the authors did not observe any significant change in the phase transition up to the highest P : L ratio studied. Our results for LFampin 265 – 284, LFampin 265 – 280, and LFampin 270 – 284 peptides with DMPC liposomes led to similar conclusions, whereas, for charged liposomes they showed that an interaction occurred in the DMPG and DMPC / DMPG (3 : 1) membranes. The lack of change in the thermotropic profile of DPPG was explained by Vogel *et al*,[34] as being due to the fact that the LFampin 268 – 284 peptide (charge + 5) could form electrostatic interactions only at the polar head level, without significantly altering the packing of the chains. A reasonable explanation for the differences between the two studies could be found in the membrane composition (DMPC vs. DPPC). DPPC had a much higher transition temperature (around 44°C) than the DMPC and the phase transition gel to *liquid-crystalline* was much more cooperative (particularly in the MLVs), due to the higher hydrocarbon chain length of the DPPC, which led to additional van der Waals interactions. This had to be responsible for a lower partition of the peptide to this model membrane system.

Ladokhin and White[43] studied the interaction profiles of melittin with zwitterionic and anionic model membranes. The authors suggested that, unlike with PC membranes, melittin should not adopt a trans-membrane configuration when interacting with anionic liposomes (PG), and that the permeabilization of these later membranes by melittin was possibly due to a mechanism of ‘leaky fusion’. These authors also showed that the mechanism of permeabilization of the membrane was not an inherent characteristic of the peptide, but strongly depended on the nature of the lipid bilayer. Epand *et al*, had proposed that cationic peptides (α/β peptides) and polymers that mimic antimicrobial peptides could segregate the anionic lipids from mixed membranes, forming rich negative lipid / peptide domains, causing defects in the membrane with consequent loss of internal content.[44–46] The observation of domain formation between the cationic peptide and negative lipid membrane had been proposed earlier by McLaughin and colleagues.[47] They studied the interaction between MARCKS and pp60^Src^ (mimetic peptides from the charged region of the protein as phospholipase C and kinase C) with mixed phospholipid membranes of PC / PG and PC / PS, and found that the strength of the interaction was influenced by the cationic amino acid residues content and by the anionic lipid fraction in the membrane. Furthermore, they reported that for the peptides studied, the electrostatic interaction was independent of the nature of the anionic lipid (PS or PG) and of the cationic amino acid residue (lysine or arginine). The authors interpreted the sigmoid shape of their binding curves as a function of the negative lipid fraction in the membrane, and as resulting from the peptide-induced formation of domains rich in negative lipid. The formation of domains with negative lipids was also reported by Lohner *et al*,[48] for the peptide PGLa.

Our results for the biophysical characterization of the interaction of peptides with model membrane systems were also in very good agreement with the ones we obtained for antimicrobial activity (against *E. coli, S. sanguinis, and C. albicans*), even considering the simplicity of the model membranes used, where we only introduced the lipid DMPG for modeling the pathogens, as it was well known to be one of their major components. The peptide with the highest antimicrobial activity was LFampin 265 – 284, followed by LFampin 265 – 280, whereas, LFampin 270 – 284 proved to be inactive against the tested microorganisms [Figure 2]. Our CD results indicated that LFampin 265 – 284 had the highest percentage of α-helix in the presence of negatively charged membranes, followed by LFampin 265 – 280, whereas, LFampin 270 – 284 remained unstructured in the presence of all the studied membranes. This confirmed the importance of a secondary structure on the antimicrobial activity, due to different interactions with the bacterial membrane. The amino acid composition also had a strong reflection in the interactions with membranes. The differences found for LFampin 265 – 284 and LFampin 265 – 280 peptides confirmed that the presence of some amino acids altered the structural arrangement of the peptide and influenced their behavior in the presence of membranes, as well their antimicrobial activity. Furthermore, as discussed earlier, both these peptides were capable of forming an α-helix in the presence of negatively charged membranes, albeit to a different extent. This was reflected in the microbiological results, in the much higher dose needed for LFampin 265 – 280 to produce the same effect as LFampin 265 – 284. The initial peptide / liposomes interaction was caused by the electrostatic attraction between the negative lipid and positively charged peptide, but the degree of interaction was differentiated by secondary structure propensities and by the amphipathicity of the peptide. As such, the secondary structure seemed to be more important than the peptide charge on peptide / pathogen (as well as peptide / membrane) interactions. Nevertheless, there was an optimum balance between charge and secondary structure (LFampin 265 – 284 vs. LFampin 265 – 280).

It should be stressed that the excellent correlation obtained between the results derived from studies with mimetic membranes and the ones obtained in vitro against different pathogens and erythrocytes[21] confirmed that biophysical experiments could be used in the initial screening of new peptides, helping in the design of new and more active drugs.
